# Micro-Scale Deformation Aspects of Additively Fabricated Stainless Steel 316L under Compression

**DOI:** 10.3390/ma17020439

**Published:** 2024-01-17

**Authors:** Abdulaziz Kurdi, Ahmed Degnah, Thamer Tabbakh, Husain Alnaser, Animesh Kumar Basak

**Affiliations:** 1 The Center of Excellence for Advanced Materials and Manufacturing, King Abdulaziz City for Science and Technology, P.O. Box 6086, Riyadh 11442, Saudi Arabia; 2 Advanced Materials Technology Institute, King Abdulaziz City for Science and Technology, P.O. Box 6086, Riyadh 11442, Saudi Arabia; adegnah@kacst.edu.sa; 3 Microelectronics and Semiconductors Institute, King Abdulaziz City for Science and Technology, P.O. Box 6086, Riyadh 11442, Saudi Arabia; ttabbakh@kacst.edu.sa; 4 Material Science and Engineering Department, University of Utah, 135 S 1460 E, WBB 112, Salt Lake City, UT 84112, USA; u0685947@utah.edu; 5 Adelaide Microscopy, The University of Adelaide, Adelaide, SA 5005, Australia

**Keywords:** additive manufacturing, laser powder bed fusion, in situ compression, micro-pillar, microstructure, stainless steel 316L

## Abstract

The deformation aspects associated with the micro-mechanical properties of the powder laser bed fusion (P-LBF) additively manufactured stainless steel 316L were investigated in the present work. Toward that, micro-pillars were fabricated on different planes of the stainless steel 316L specimen with respect to build direction, and an in situ compression was carried out inside the chamber of the scanning electron microscope (SEM). The results were compared against the compositionally similar stainless steel 316L, which was fabricated by a conventional method, that is, casting. The post-deformed micro-pillars on the both materials were examined by electron microscopy. The P-LBF processed steel exhibits equiaxed as well as elongated grains of different orientation with the characteristics of the melt-pool type arrangements. In contrast, the cast alloy shows typical circular-type grains in the presence of micro-twins. The yield stress and ultimate compressive stress of P-LBF fabricated steel were about 431.02 ± 15.51 − 474.44 ± 23.49 MPa and 547.78 ± 29.58 − 682.59 ± 21.59 MPa, respectively. Whereas for the cast alloy, it was about 322.38 ± 19.78 MPa and 477.11 ± 25.31 MPa, respectively. Thus, the outcome of this study signifies that the AM-processed samples possess higher mechanical properties than conventionally processed alloy of similar composition. Irrespective of the processing method, both specimens exhibit ductile-type deformation, which is typical for metallic alloys.

## 1. Introduction

Recently, additively manufactured components are gaining applications in various real-life scenarios, which is causing a surge in additive manufacturing-related research. The improvement of the process parameters of various additive manufacturing (AM) processes on metallic alloys has been reported in recent literature [1]. Additive manufacturing is completely different to subtractive manufacturing [2]. Therefore, components made out of additive manufacturing offer different microstructural aspects. Among various AM processes, laser powder bed fusion (L-PBF) [3] is broadly used in the fabrication of metallic materials and alloys. In this procedure, a thin layer (in the range of micro-meters) is spread out on a platform, which is followed by the consolidation of the powder with the help of laser energy. As the process continues, layer-by-layer deposition gives rise to a complete three-dimensional structure.

Among various structural engineering materials, stainless steel 316L (SS 316L) is popular due to its diverse applications. In addition to that, it possesses good mechanical properties, which includes the ease of formability. The traditional way of making components out of SS 316L is casting, followed by subtractive manufacturing, as required. In contrast to that, AM of SS 316L offers a single-step fabrication process to attain near net shape components of intricate designs [4]. Moreover, the AM process itself conserves material [5]; thus, it avoids wasting material, which is not possible in subtractive manufacturing.

In general, the microstructure of the additively manufactured specimens contains characteristic melt-pool-type microstructures, where equiaxed grains exist near the melt-pool region, and somewhat columnar grains are predominant away from the melt-pool boundaries [6,7,8,9]. The microstructure of the additively manufactured SS 316L is no exception to that. Having said that, in terms of grain size development, AM has a limited role on grain refinement [6,10]. These features were attributed to the “directionality of solidification”, as well as the “build-direction”, due to prevailing higher cooling rates compared to traditional casting processes [11,12]. Obviously, these unique microstructural developments play a significant role in their mechanical properties. As reported by Kurdi et al. [8], additively manufactured SS 316L exhibits about one and a half times higher hardness than their wrought counterpart (1.92–2.12 GPa vs. 1.30 GPa). In addition to that, “the plasticity resistance of L-PBF alloy was about 1.15 times higher than the wrought alloy, and contributed towards higher shear stresses of the L-PBF alloy (274.5–294.4 MPa), compared to 175.95 MPa for the wrought alloy”. The main consequence of AM on the mechanical properties of the materials is the introduction of anisotropy. The effect of such anisotropy on the strength of the material was also reported by Guan et al. [11] on their selective laser fabricated 304 stainless steel. The effects of building direction on the mechanical properties were obvious, as the vertical built test specimen (90°) exhibited the optimized combination of strength and ductility. Moreover, specimens made with 20 µm layer thickness offered the highest yield strength (530–551 MPa), ultimate tensile strength (696–713 MPa) and elongation (32.4–43.6%), while the specimen with 30 µm layer thickness offered the lowest values for all tensile properties. There was no obvious difference in the tensile properties for the test specimens with 20, 30 and 40 µm layer thickness, and the reason behind that is the similar nature of the microstructure and metallurgical bonding. According to Liverani et al. [12], the impact of laser power on elongation to failure, yield, and ultimate tensile strength was negligible on their investigated L-PBF 316L austenitic stainless steel. Having said that, the yield strength increased from 10% to 20%, when the orientation angle change from 45° to 90°, irrespective of laser power (100 W and 150 W). According to their statement, building orientation had considerable effects on the corresponding mechanical properties, whereas the applied laser power and hatch spacing played a minor role. Itziar et al. [13] studied the mechanical properties and the variability of the manufacturing orientation of ASIS 316L stainless steel processed by selective laser melting (SLM). The mechanical properties of the final product were then compared with the wrought (cast and forged) products. According to their findings it could be summarized that irrespective of the orientation of the specimens, the yield strength of the additively manufactured specimens is always higher than that of wrought alloys by keeping similar elongation values. An increase in the ratio of tensile strength against the yield strength would be able to save additional material; freedom of design of test specimen and weight drops could be achieved by the additive manufacturing process involved, but this caused a reduction in elongation. According to their reported results, the ratio of tensile strength against yield strength was higher than that for the wrought product. For the L-BBF specimen, the ultimate tensile strength, yield strength and percentage elongation values were 685–691 MPa, 662–678 MPa and 25–33%, respectively. In contrast, the wrought alloy exhibits 520–680 MPa of ultimate tensile strength, 220–270 MPa of yield strength and 40–45% elongation. Similar results were published by other researchers: for example, Li et al. [14] and Vittoria et al. [15] on austenitic stainless steel. In view of that, the present work is the continuation of our previous work [8], where the indentation-related mechanical properties and deformation aspect were reported. The novelty/significance of the present work is the in situ compression of micro-pillars to access the micro-mechanical properties and deformation behavior of AM-processed alloy at a micro-scale.

In view of that, the present research aims to study the deformation aspect on L-PBF stainless steel 316L at the micro-scale. This was achieved by micro-pillar compression inside of an SEM chamber, and in addition to that, micro-mechanical properties of the alloy were also attained. In addition to that, the anisotropic aspect of the mechanical properties was also investigated. The outcomes of the present work will develop the fundamental understanding on how such an alloy deformed under mechanical loading.

## 2. Materials and Methodology

### 2.1. Feed-Stock Powder and Fabrication of Specimens

The gas-atomized feed-stock powder of the stainless steel 316L was procured commercially from Valimet Inc., Stockton, CA, USA. The powder composition (elemental) was 16–18 wt. % Cr, 10–14 wt. % Ni, 2–3 wt. % Mo, 2 wt. % (max.) Mn, 1 wt. % (max.) Si, 0.045 wt. % (max.) C, and the balance was Fe. As reported in our previous communication [8], the powder particles are in a spherical shape, which varies in the range of 20–60 µm. The additive manufacturing unit was a SLM 250 HL system (SLM Solutions Group, Germany) with maximum power output capacity of 400 W from continuous waves of an Nd:YAG laser. The following printing parameters were employed, as reported in our previous study [8]: “320 W of laser power, 0.1 mm of hatch distance, 0.05 mm of layer thickness, and 600 mm/s of scan speed”. These parameters were carefully chosen in view of the data stated in the literature and the ‘trial and error’ optimization process to obtain dense specimens. According to the literature, the energy density (ED) of the process is [16]: ED = P/(v_s_·h·t), where P is laser power, v_s_ is speed, h is hatch distance, and t is the thickness of the powder layer. To uphold an energy density of 62.5–104.2 J/mm^3^ [17,18], the following input parameters were selected: 320 W of laser power, 0.1 mm of hatch distance, 0.05 mm of layer thickness, and 600 mm/s of scan speed. To limit the oxidation of the constituent elements, Ar was purged in the closed loop system. A 67° scanning strategy was employed to reduce stress build-up together with post stress relief [19] at 240 °C for 1 h duration.

**Figure 1:**
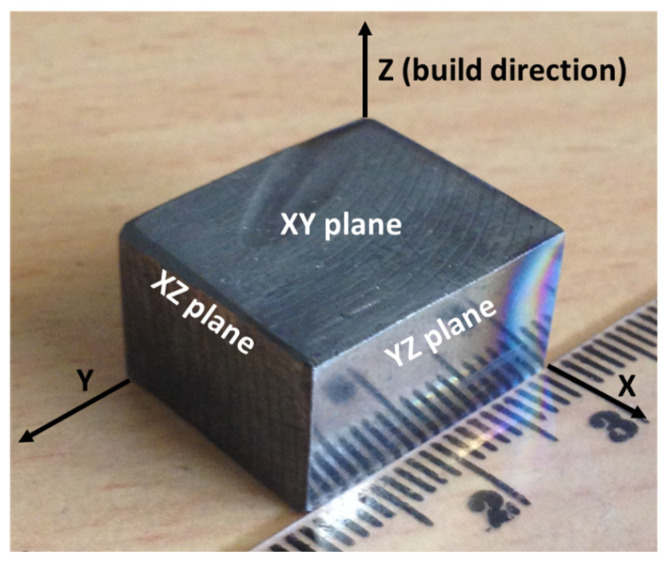
As fabricated block of stainless steel 316L fabricated by L-PBF process. The direction of build (z) is also indicated in the figure.

As reported in our previous communication [8], the “Archimedes principle was applied to evaluate the density of the as-built samples. Stainless steel 316L of similar composition, however, processed by traditional casting (and forging) was also obtained commercially (Rolled Alloys Ltd., Singapore), and used as a reference material, subjected to identical testing. To investigate the anisotropic aspect [20], if there are any, both microstructural and mechanical investigations were conducted on different planes of the rectangular block specimen, and termed accordingly. The plane that is vertical to the build direction is termed as the horizontal (XY) plane, whereas the planes that are parallel to build direction are denoted as frontal (XZ) and lateral (YZ) planes. The appearance of the as-built sample, together with different planes are shown in Figure 1”.

### 2.2. Metallography of the Specimens and Scanning Electron Microscopy (SEM)

The as-fabricated block of the specimen was cut in half in the middle and mounted in a resin block by a hot-mounting process (Cito press-10, Struers, Ballerup, Denmark). Then, the blocks were grinded and polished in polishing cloths with varying polishing slurry in a “Struers automatic metallographic polisher”. The final polishing was conducted in colloidal silica, and the polished surfaces were mirror-like and scratch-free. As per the nominal procedure of specimen characterization, the microstructures of the samples were investigated in backscattered electron (BSE) mode in a field emission scanning electron microscope (Helios Nanolab 600 FIB-SEM, Thermo Fisher Scientific, Portland, OR, USA). The elemental analysis was conducted by Oxford X-ray spectrometry (EDS) (London, UK) attached with the SEM [8]. The same equipment was used to fabricate the micro-pillar by employing ion beam milling. For BSE imaging, the SEM was operated at 10 KV with 0.34 nA current. EDS analysis was carried out under the same 10 KV setting, however, with higher current, i.e., 1.4 nA for better collection efficiency of the signal.

### 2.3. Micro-Pillar Compression

Compression experiments on micro-pillars were conducted inside an SEM, where the Hyistron PI-88 (Bruker, Billerica, MA, USA) indentation system was mounted on the SEM stage. The compression was conducted in ‘displacement control mode’, and the loading rate was 3 nm/s, which corresponds to a 10^−3^ s^−1^ strain rate. The morphology of the compressed micro-pillars was also examined by the SEM. The output of the micro-pillar compression was the load–displacement graphs. These load–displacement graphs were used to calculate the stress–strain curves, according to the procedure proposed by Misra et al. [21], which is widely accepted. A minimum of 6 individual micro-pillars were compressed in each case, and average values were reported together with standard deviation as statistical analysis.

## 3. Results and Discussion

### 3.1. Microstructural Analysis

**Figure 2:**
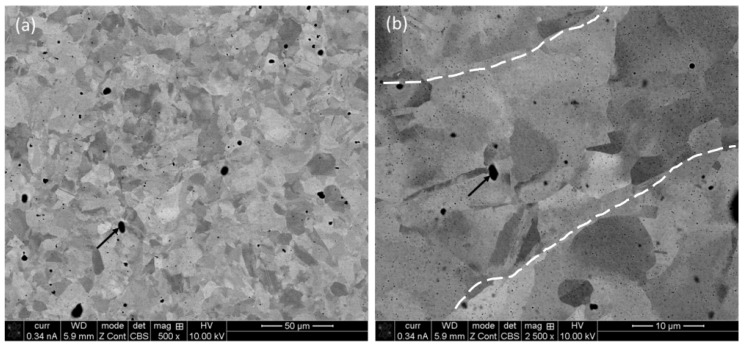
Backscattered electron image (BSE) of P-LBF SS 316L sample on horizontal plane at low (×500) (**a**) and high (×2500) (**b**) magnification exhibiting the morphology of the grain evolution.

Figure 2 displays the BSE micrographs of the horizontal plane (XY) of the specimen at low (×500) and high (×2500) magnifications. The low magnification micrograph (Figure 2a) exhibits the overall microstructural evolution of the specimen, i.e., grains of different size and orientation, together with pores (as indicated by black arrows). The back-scattered mode of SEM imaging is dominated by elemental contrast, indicating an absence of any elements, i.e., pores that appear as dark spots. These features are more prominent in high magnification micrographs (Figure 2b) in addition to the existence of boundaries of the melt-pool, as marked out with dotted lines in Figure 2b.

**Figure 3:**
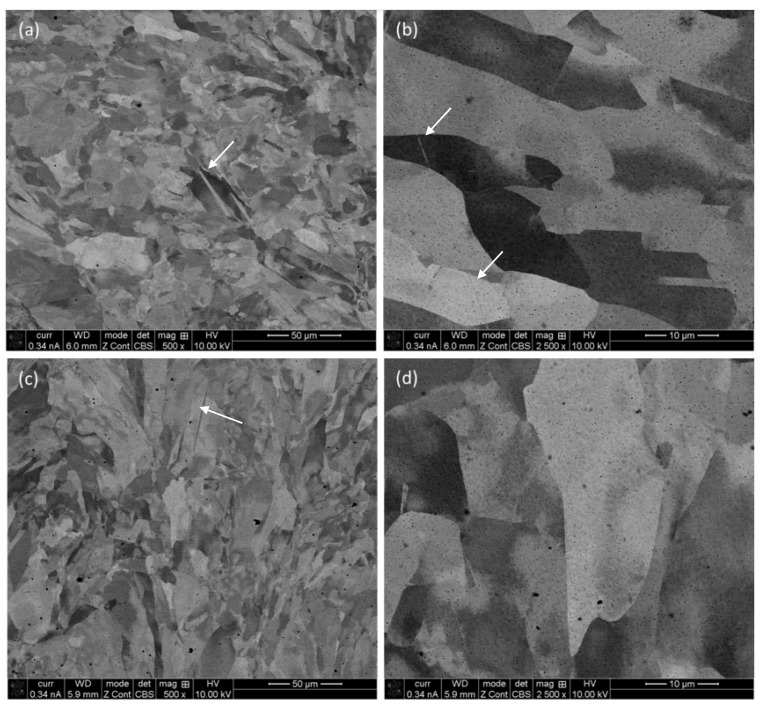
Backscattered electron image (BSE) of L-PBF SS 316L sample on the frontal (**a**,**b**) and lateral (**c**,**d**) planes at low (×500) (**a**,**c**) and high (×2500) (**b**,**d**) magnifications, exhibiting the morphology of the grain evolution.

The origin of the contrast of different grains originates from their respective crystallographic orientation, as stated in our previous publications [8], in view of the electron backscattered diffraction (EBSD) analysis. Similarly, the microstructure of the specimen in frontal and lateral planes at different magnifications is shown in Figure 3. Analogous to what was observed in the micrographs on the horizontal plane, there are grains of different size and orientation together with pores spread throughout the microstructure. In contrast to the equiaxed outlook of the grains in the horizontal plane (Figure 2), the grains on “the lateral and frontal planes (Figure 3) are elongated in nature”. This is due to the fact that the L-PBF process favors the growth of the grains along the build directions, which eventually cohabitate in the direction of maximum thermal gradient [22]. The thermal gradient drives the grains to grow over multiple layers (thickness of the powder bed), and after solidification, the grains seem elongated from lateral and frontal planes viewpoints.

As mentioned before, the pores are present all throughout the specimen, as evident from the micrographs of three different viewpoints (horizontal, frontal and lateral planes). These pores are metallurgical in nature and form due to gas entrapment [23,24]. Thus, the process parameters need to be refined to limit/eliminate the amount of such pores. The usual approach is post-heat treatment [25,26,27]. However, post-heat treatment is susceptible to grain growth, and it can nullify the effect of grain refinement, which was achieved through the L-PBF fabrication process. Thus, it is necessary to come out with some new innovative approach. In addition to that, the existence of some nano-twins was also confirmed (as marked in Figure 3 with white arrows). As the aim of the present manuscript was to compare the compressive mechanical properties of the L-PBF specimen to that of wrought alloy of similar comparison, post-heat treatment of the L-PBF processed specimens was avoided.

**Figure 4:**
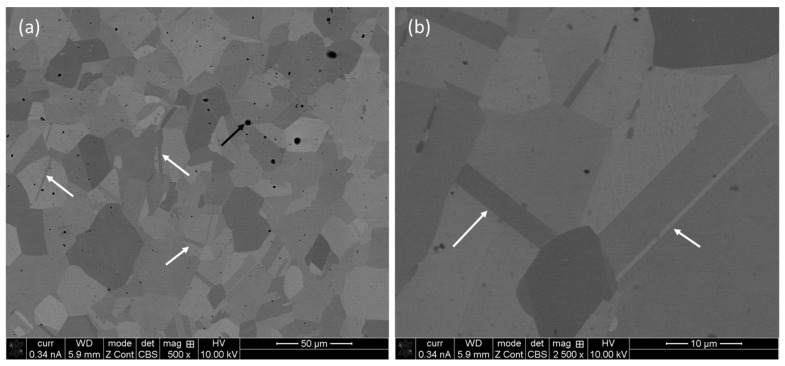
(**a**) Backscattered electron image (BSE) of wrought SS 316L at low (×500) and (**b**) high (×2500) magnifications. The arrows indicate the location of twins.

To have a comparison with the microstructure of a wrought alloy of similar composition, Figure 4 depicts the micrographs of a commercially acquired wrought alloy of SS 316L fabricated by casting, which was followed by forging. The grains are marginally larger (average grain size of 3.6 µm) to that of the L-PBF processed alloy (average grain size of 3.2–3.4 μm), as demonstrated in our previous publication [8]. This was attributed to the relatively lower solidification rates of the casting process compared to that of the L-PBF process, which favors the growth of equiaxed grains of larger diameter. Again, the contrast of the grains is due to the orientation of the grains in different directions. There are also metallurgical pores, which are common casting defects [28]. Together with that, the twins are abundant, which are of micro-scale size, in contrast to nano-scale twins in L-PBF processed alloys.

**Figure 5:**
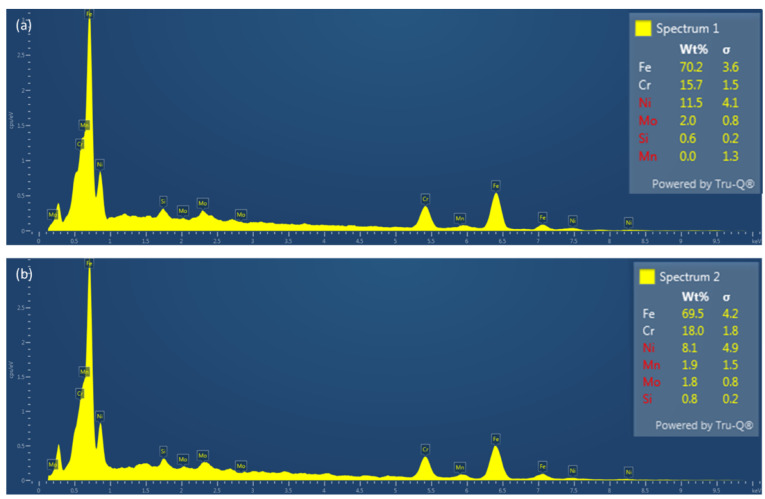
EDX spectra on (**a**) L-PBF and (**b**) wrought alloy, confirming the elemental composition of the currently investigated specimens.

The elemental analysis of the both L-PBF and wrought alloys is shown in Figure 5. The minute difference in composition is mostly within the range of experimental error. Thus, it can be stated that there was no loss of certain elements in the course of L-PBF for this particular alloy, unlike certain other alloy systems [29].

As reported in our previous report, the density of the currently examined L-PBF processed alloy is 97.3% to that of theoretical density [30], whereas for the wrought alloy, it is about 99.6%. The density of the L-PBF processed alloy is in the upper range to that reported in the literature [31], thanks to the use of optimum process parameters.

### 3.2. Micro-Pillar Fabrication and Compression

**Figure 6:**
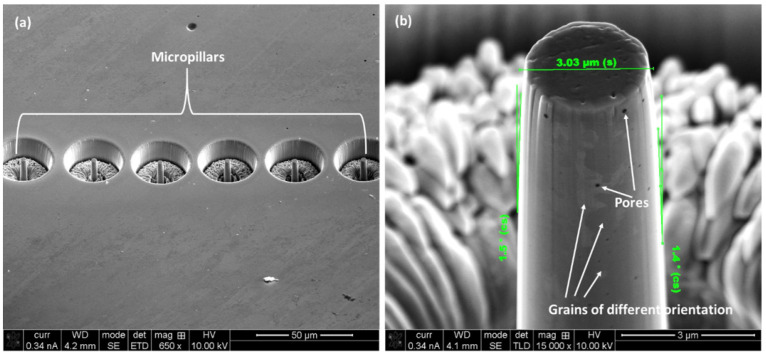
(**a**) A number of micro-pillars on the horizontal plane of the L-PBF SS 316L sample together with high magnification image (**b**).

At first, the micro-pillars were made on the polished samples with the help of FIB-SEM. The dimensions of the micro-pillars were 3 μm in diameter, with 9 μm of length, thus maintaining an aspect ratio of 1:3. Enough volume of material (30 μm diameter) around the micro-pillar was removed so that the indenter does not touch the bulk material and induce an artefact in the results. At the beginning, 9.6 nA current at 30 kV was employed to accelerate the removal of material in the ‘angular milling’ mode. After that, a lower amplitude current was employed gradually to obtain a smooth pillar surface “with final polishing at 93 pA at 30 kV. A representative SEM micrograph of a series of micro-pillars made on horizontal plane of the L-PBF sample are shown in Figure 6a, together with a higher magnification image in Figure 6b. As evident from Figure 6b, the micro-pillars are slightly taper (<2°), which is unavoidable, due to the interaction of the ion beam with the materials [14]”. In addition to that, the orientation of different grains and the presence of pores are also evident, as pointed out by the arrows. This also confirms that a given micro-pillar contains several grains of different orientation in it, which makes the micro-pillar a true representation of the bulk material. At least five independent compression tests were conducted for a given scenario to achieve sound statistical analysis, and average data were stated for the results and discussion.

### 3.3. Stress–Strain Curves of Compression

**Figure 7:**
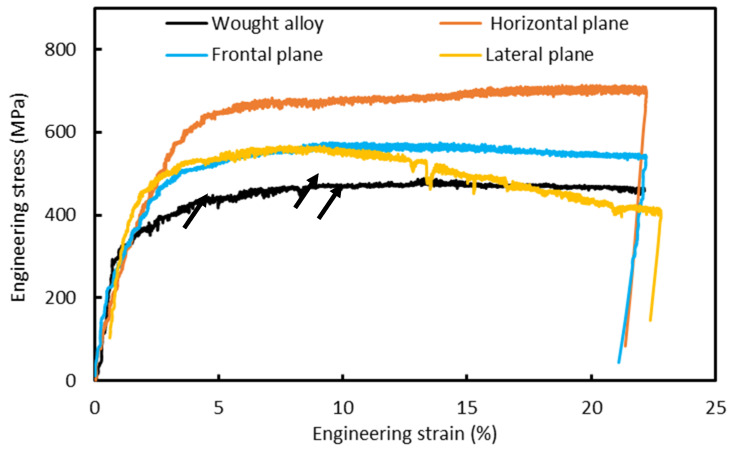
Characteristic stress–strain graphs obtained on L-PBF processed and wrought SS 316L. The black arrows indicate the sudden drops in stress.

As stated earlier, load–displacement curves obtained during micro-pillar compression were converted into stress–strain curves, as shown in Figure 7. During calculation, the applied force was divided with the area by taking consideration of Sneddon’s effects [21]. Detail equations and calculation procedures are available in the literature [21], and therefore were avoided here. At first glance, the stress–strain curves show the typical behavior of a ductile metallic alloy. At the first stage, there is a sharp rise of stress with a corresponding level of strain, followed by a plateau, where the strain continues to rise at a given range of stress. This represents the ductility behavior of the material, which is quite predominant in the present case. As also evident from Figure 7, the L-PBF alloy outperformed the wrought alloy both in terms of yield stress and ultimate compressive stress. The yield stress of the L-PBF alloy is in the range of 431.02–474.44 MPa, whereas for the wrought alloy, it is about 322.38 MPa. Similarly, the ultimate compressive stress of the L-PBF alloy is 547.78–682.59 MPa, whereas it is about 477.11 MPa for the wrought alloy. In addition to that, the elastic modulus of the alloys was also calculated from the linear portion of the stress–strain curves [32] and reported in Table 1 together with other mechanical properties. Now, within the same alloy, the horizontal plane exhibits comparatively higher mechanical properties (474.44 MPa of higher yield strength and 682.59 MPa of ultimate compressive strength) to those of the frontal (431.02 MPa of yield strength and 561.63 MPa of ultimate compressive strength) and lateral planes (444.82 MPa of yield strength and 547.78 MPa of ultimate compressive strength). As the only difference among different planes is the microstructure (i.e., the orientation of different grains), thus, these differences in the mechanical properties must be due to this.

**Figure 8:**
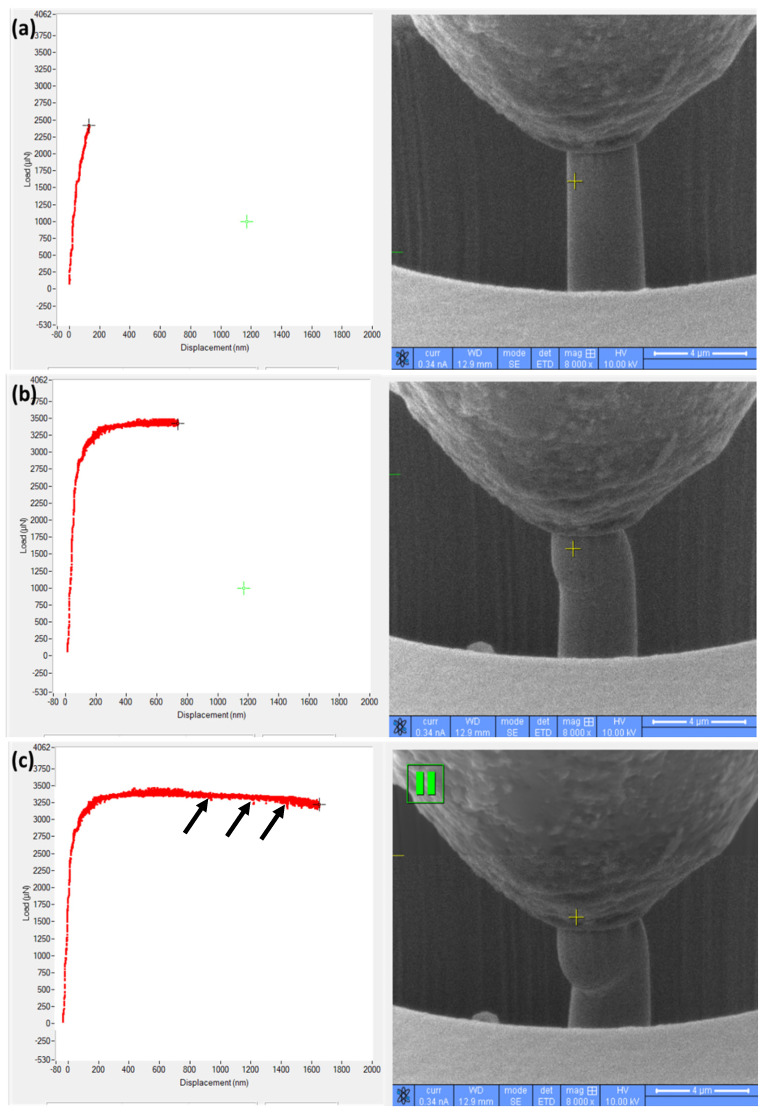
Physical outlook of the compressed micro-pillar with corresponding load–displacement curve at the given interval: (**a**) beginning, (**b**) middle and (**c**) just before the completion of the compression experiment. The black arrows indicate the sudden drops in stress.

As also evident from Figure 7, irrespective of the alloys and planes, there are sudden drops in stress for the given strain level, as indicated with black arrows in Figure 7. This is a typical characteristic outcome of the micro-pillar compression, and it is due to the initiation/movement of the slip and shear band, as described in the literature [8]. This was also the scenario in the present case, as verified from the recorded videos of the micro-pillar compression. An example of that is shown in Figure 8 (from the screenshot of the recorded video), where the physical state of the micro-pillar at various strain levels is shown together with the position of corresponding points in the stress–strain graphs. Figure 8 represents the compression on the horizontal plane of the L-PBF alloy. As shown in Figure 8a, there was no visible physical deformations during the linear portion of the stress–strain curve, as the deformation was mostly elastic in nature. After that linear portion, initiation of the slip/shear planes took place, leading to an onset in the yielding of the material, i.e., the plastic deformation. These slip/shear planes multiply in numbers (Figure 8b), in all directions of the micro-pillar, as the compression continues. Sometimes, a sudden drop in stress occurs due to the rearrangement of the contact between the crumbling micro-pillar and the indenter itself, as indicated with black arrows in Figure 8c. These scenarios continue (Figure 8c) until the completion of the experiment, as the materials in the present case are fully ductile in nature. As stated by Basak et al. [33], “Once the applied load exceeds the critically resolved shear stress [18], the crystal structure cannot rearrange anymore, and the formation of micro-voids take place, followed by the plastic flow of the materials, along with favoured slip/shear planes”. These suggest that there exists an anisotropy in the mechanical response of the L-PBF alloy, as stated in the literature [34]. This is due to the fact that the loading direction is parallel to the slice layers but perpendicular to the columnar grains for the horizontal specimen, and it represents the highest tensile strength.

### 3.4. Morphology of the Compressed Micro-Pillars

**Figure 9:**
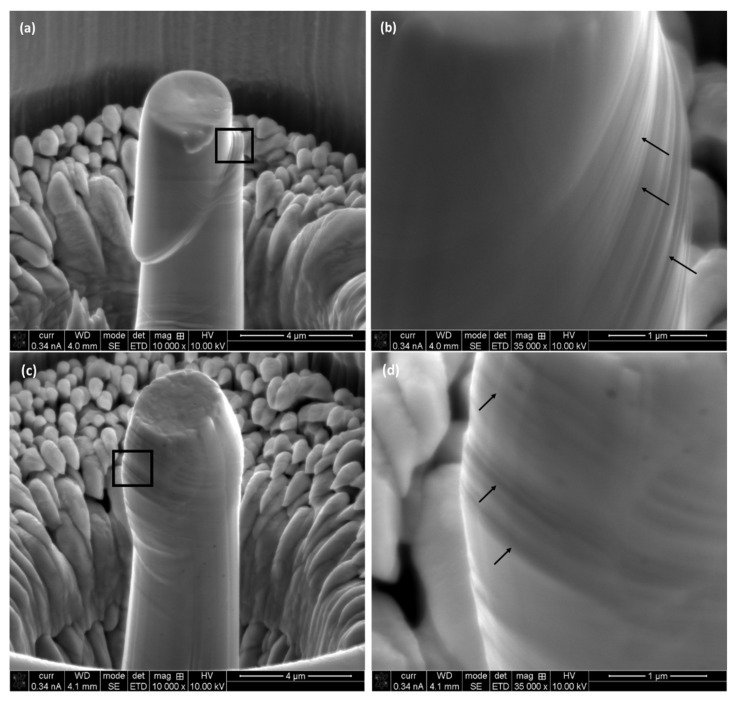

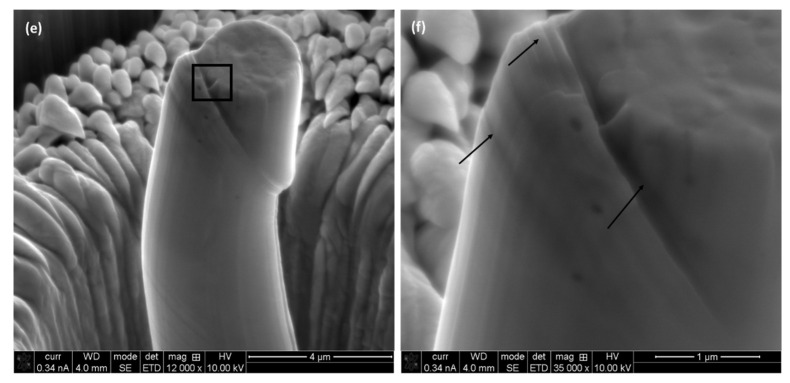
Outlook of the deformed micro-pillars on different planes of L-PBF processed alloy: (**a**,**b**) horizontal, (**c**,**d**) frontal and (**e**,**f**) lateral planes. Higher magnification (×35,000) images of the marked areas (**a**,**c**,**e**) are shown next to it (**b**,**d**,**f**).

**Figure 10:**
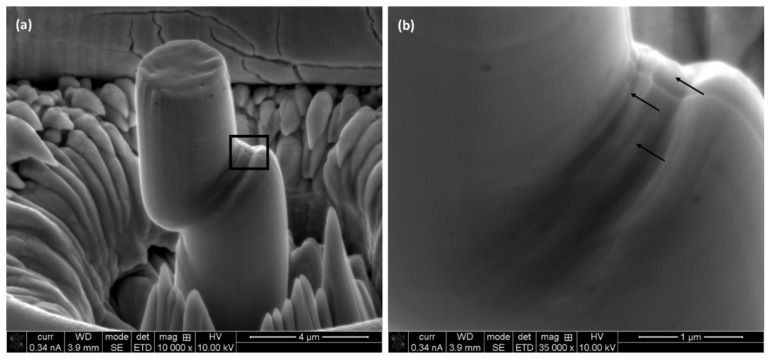
Outlook of the deformed micro-pillars on wrought SS 316L higher magnification (×35,000) images of the marked area (**a**) is shown next to it (**b**).

Upon compression, the morphology of the crumbled micro-pillars was investigated closely with the help of SEM, as shown in Figure 9 and Figure 10. Figure 9 shows the outlook of the micro-pillars on L-PBF process alloys on different planes at 10,000 times magnification (Figure 9a,c,e). Most of the deformation is confined in the top section of the micro-pillar, which is infested with slip planes and shear bands. As shown in the higher magnification (×35,000) images (Figure 9b,d,f), the areas between slip planes/shear bands are ductile in nature without the formation of any visible voids at the macro-scale.

Similar to that, the wrought alloy also exhibits ductile mode dominated deformations, as shown in Figure 10. Unlike the L-PBF processed alloys, the main deformation took place somewhat in the middle of the micro-pillar (Figure 10a). Again, the deformation aspect is fully ductile in nature, as confirmed by the continuation of the material among the ridges of the slip/shear planes, which is due to plastic flow.

## 4. Conclusions

The micro-mechanical aspects of SS 316 L alloy fabricated by L-PBF and a wrought process was investigated in this study along with their deformation outlook. The microstructure of the L-PBF processed SS 316L alloy shows the typical microstructure of other L-PBF fabricated metallic alloys, i.e., the formation of melt-pool boundaries, along with the presence of equiaxed gains near the melt pool boundaries, and somewhat columnar (elongated) grains, away from the melt pool boundaries. The prevalence of anisotropy in the microstructure was also evident, which affected its mechanical properties and gave rise to anisotropy in mechanical behavior. The horizontal plane exhibits a higher yield strength (474.44 MPa) and ultimate compressive strength (682.59 MPa) compared to that of the lateral (444.82 MPa of yield strength and 547.78 MPa of ultimate compressive strength) and frontal lateral (431.02 MPa of yield strength and 561.63 MPa of ultimate compressive strength) planes. Compared to that, the yield strength (322.38 MPa) and ultimate compressive strength (477.11 MPa) of wrought alloy falls behind. Thus, it could be stated that the AM processing technique holds the potential to enhance the mechanical properties of the alloys by modulating microstructural evolution compared to that of casting. In respect to the deformation outlook, both L-PBF and wrought alloy exhibited similar behaviors, which is the initiation and propagation of shear and slip bands, and this leads to the ultimate failure of the material.

## Figures and Tables

**Table 1:** Mechanical properties of the currently investigated alloys.

Mechanical Properties	P-LBF Fabricated SS 316L			Cast (Wrought) SS 316L
	Lateral Plane	Horizontal Plane	Frontal Plane	
Yields stress (GPa)	444.82 ± 21.45	474.44 ± 23.49	431.02 ± 15.51	322.38 ± 19.78
Ultimate compressive stress (GPa)	547.78 ± 29.58	682.59 ± 21.59	561.63 ± 27.56	477.11 ± 25.31
Young’s modulus (GPa)	237.82 ± 22.51	221.26 ± 17.59	214.03 ± 25.11	251.37 ± 10.59

## Data Availability

The raw/processed data used to produce the results will be made available by the corresponding author upon reasonable request.
